# Respiratory infections in children: an appropriateness study of when parents should home care or seek medical help

**DOI:** 10.3399/bjgp20X713933

**Published:** 2020-12-15

**Authors:** Louise Newbould, Stephen M Campbell, George Edwards, Rebecca L Morris, Gail Hayward, Emma C Hughes, Alastair D Hay

**Affiliations:** Social Policy Research Unit, University of York, York.; National Institute for Health Research Greater Manchester Patient Safety Translational Research Centre, School of Health Sciences, Manchester Academic Health Science Centre, University of Manchester, Manchester.; Nuffield Department of Primary Care Health Sciences, University of Oxford, Oxford.; National Institute for Health Research Greater Manchester Patient Safety Translational Research Centre, School of Health Sciences, Manchester Academic Health Science Centre, University of Manchester, Manchester.; Nuffield Department of Primary Care Health Sciences, University of Oxford, Oxford.; Manchester University NHS Foundation Trust, Manchester.; Centre for Academic Primary Care, NIHR School for Primary Care Research, Bristol Medical School; Population Health Sciences, University of Bristol, Bristol.

**Keywords:** consensus, general practice, healthcare utilisation, primary health care, respiratory tract infections

## Abstract

**Background:**

Children with respiratory tract infections (RTIs) use more primary care appointments than any other group, but many parents are unsure if, and when, they should seek medical help and report that existing guidance is unclear.

**Aim:**

To develop symptom-based criteria to support parental medical help seeking for children with RTIs.

**Design and setting:**

A research and development/University of California Los Angeles (RAND/UCLA) appropriateness study to obtain consensus on children’s RTI symptoms appropriate for home, primary, or secondary health care in the UK.

**Method:**

A multidisciplinary panel of 12 healthcare professionals — six GPs, two pharmacists, two NHS 111 nurses, and two emergency paediatric consultants — rated the appropriateness of care setting for 1134 scenarios in children aged >12 months.

**Results:**

Panellists agreed that home care would be appropriate for children with ≤1 week of ‘normal’ infection symptoms (cough, sore throat, ear pain, and/or runny nose, with or without eating adequately and normal conscious level). The presence of ≥2 additional symptoms generally indicated the need for a same-day GP consultation, as did the presence of shortness of breath. Assessment in the emergency department was considered appropriate when ≥3 symptoms were present and included shortness of breath or wheezing.

**Conclusion:**

The authors have defined the RTI symptoms that parents might regard as ‘normal’ and therefore suitable for care at home. These results could help parents decide when to home care and when to seek medical help for children with RTIs.

## INTRODUCTION

Respiratory tract infections (RTIs) in children are the most common reasons for primary care consultations^1^ and antibiotic use,^2^ and are a key area for antimicrobial stewardship research.^3^ The National Institute for Health Research School for Primary Care Research (NIHR SPCR) has undertaken an analysis of 100 million consultations in England.^4^ It found *‘a substantial increase in practice consultation rates, average consultation duration, and total patient-facing clinical workload in English general practice’* .^4^ A recent survey of 901 GP surgeries found the average waiting time for a GP appointment to be >2 weeks^5^ and that *‘English primary care could be reaching saturation point’.*^4^ There is an urgent need for radical change and for a partnership between service providers and patients in using health and social care appropriately.

There is considerable parental uncertainty regarding if, and when, to consult NHS services when children fall ill with RTIs.^6^^,^^7^ NHS data show a tenfold between-GP-practice variation in RTI consultation rates.^2^ Parents may not possess sufficient understanding regarding the point at which it is appropriate to consult a GP,^8^ with around 70% of parents consulting within 1 week of symptoms presenting,^9^ many for reassurance.^10^^,^^11^ Parents use a variety of different sources of information about help seeking (for example, from friends and family, websites with advice, or leaflets) but report that they are often confusing and unhelpful.^11^ In part, this may be because these sources are influenced by the medicolegal need to detect the small proportion of children with a serious life-threatening illness.^12^

Safer, cost-effective, and more pragmatic interventions are needed to help parents make appropriate use of scarce primary care resources^4^ with clear and relevant advice for parents. The aim of this study was to develop symptom-based criteria that parents could use when considering how to respond to a child who has had respiratory infection symptoms for ≤1 week.

## METHOD

### Overview of the RAND/UCLA Appropriateness Method

The research and development/University of California Los Angeles (RAND/UCLA) Appropriateness Method (RAM) is used to help develop guidance on responses to healthcare scenarios, especially where ‘robust scientific evidence’ is lacking or contested.^13^

**Table:** How this fits in

Demand for health care is increasing unsustainably. Parents report existing advice regarding when to consult for a child with respiratory infection symptoms to be confusing and unhelpful. The present study showed that children with ≤1 week of cough, sore throat, ear pain, and/or runny nose, with or without eating adequately and normal conscious level, can be regarded as ‘normal’ and suitable for home care. Results could improve child and parent healthcare experience by providing a clear and evidence-based information source on appropriate help-seeking behaviour, while optimising the appropriate use of hard-pressed healthcare services.

### Development of criteria

The RAM scenarios were developed iteratively by identifying and prioritising scenarios based on best practice;^9^^–^^11^^,^^14^^–^^16^ these were then compared with existing evidence (G Edwards *et al*, unpublished data [2020]) and verified by three clinical academics in general practice and paediatric emergency medicine.

First, a clinical ‘stem’ was devised, describing common symptoms that the ‘average’ child with a normal respiratory infection might have: *‘up to a week of respiratory infection symptoms (for example, cough, sore throat, and/or runny nose) with or without eating adequately and normal conscious level’*. Next, two key variable parameters were identified: first, the child’s age — (a) 12–23 months; (b) 24 months–4 years and 11 months; and (c) 5–12 years); and, second, their past medical history — previously well aside from usual childhood illnesses, with or without asthma; or a previous admission for asthma, bronchiolitis, or other respiratory condition. Six symptoms were then prioritised as of concern to parents: ear pain; wheeze (defined as ‘wheeze or noisy breathing’); shortness of breath; reduced fluid intake; less active or socially interactive than usual; and high fever. Finally, three appropriate ‘next steps’ were identified for panellists to consider as the parental response to each scenario. These were: monitor, home care, go to pharmacy, or routine (non-urgent) GP appointment; seek a same-day GP (in hours) or NHS 111 (out-of-hours) appointment; or go to emergency department (ED)/telephone 999. Together, these resulted in 1134 unique scenarios.

Three clinicians, known to the last and fifth authors, provided feedback on the study documents and on the ‘stem’ and ‘symptoms’ scenarios to corroborate clinical relevance. Additionally, study documents and scenarios were then presented at a dedicated project public and patient involvement group within the NIHR Greater Manchester Patient Safety Translational Research (GMPSTR) Centre.

### Selection and description of participants

A multidisciplinary panel of 12 healthcare professionals was recruited. These professionals were recruited through snowball sampling, starting with the fifth and last authors of this study, in addition to utilising the local Clinical Research Network (CRN). The panel consisted of six GPs, two pharmacists, two NHS 111 nurses, and two hospital emergency paediatric consultants.

### RAND/UCLA appropriateness method rounds

The RAM process entailed two rounds of ratings with each panellist rating each scenario on a 9-point integer scale, where 1 rates the next step as completely inappropriate and 9 rates it as completely appropriate. Round 1 was conducted by email on a Microsoft Excel (version 2016) rating sheet in May 2019 with panellists rating each of the 1134 clinical scenarios individually, without discussion.

Round two comprised a one-day, face-to-face meeting held in June 2019. Panel members were presented with data from round one outlining the distribution of all panel members’ ratings, the median, and their own individual data from round one for each of the 1134 scenarios.^17^ The moderator raised particular areas of disagreement from round 1 and resolved any issues regarding the understanding of the rating scale.^17^ Unlike a postal or online method, the strength of the face-to-face meeting is the opportunity for real-time panel discussion and interaction. Panellists then discussed and individually re-rated each scenario on the Excel sheets.^17^ Panel members were not required to reach consensus.^17^ The panel was co-chaired by two of the authors, one an expert in RAM,^13^^,^^18^^–^^21^ and one a GP expert in RTI,^14^^,^^22^^,^^23^ neither of whom contributed to the RAM scores.

### Statistical analysis

Data from round two were collated and double entered by the first and sixth authors. These data were analysed to determine the median rating of the appropriateness of the next step (home care; GP/NHS 111; or ED/999) and the level of consensus within the panel for each scenario. Definitions for ‘agreement’, ‘disagreement’, and ‘equivocal’ were as previously published.^17^^,^^19^ Agreement was defined by at least 9 of the 12 (75%) panel members rating the same 3-point region on the 9-point integer scale, that is, 1–3; 3–5, 6–8, and so on. A proposed action was then categorised as an ‘appropriate’ next step with agreement if a scenario was rated 7–9 points (overall panel median of 7–9 points), or ‘inappropriate’ with a rating of 1–3 points with agreement (median 1–3 points). Disagreement was defined as ≥4 panellists (≥33%) rating a scenario in both the 1–3 points range and 7–9 points range on the 9-point integer scale. Where the next step was to go to ED/telephone 999, these categories were separated to dichotomise depending on urgency: an overall panel median of 7–8 points indicated taking the child to the ED would be the appropriate next step, while a median of 9 points indicated that telephoning 999 would be appropriate. Ratings of clinical scenarios without agreement or disagreement that the next step was either appropriate or inappropriate, or that had a panel median of 4–6 points with agreement, were considered equivocal.

## RESULTS

The clinical stem emphasising the presence of symptoms of RTI for ≤1 week in a child, preceded the rating of the scenarios. Box 1 shows an example of how the scenarios were presented to the panellists for rating (the symptoms highlighted in yellow were present in the scenario shown).

**Box 1.** Example scenario for rating by panellists^a^ Clinical stem: For the parent of a child aged (a) 12–23 months/(b) 24 months–4 years and 11 months/(c) 5–12 years with ≤1 week of respiratory infection symptoms, for example, cough, sore throat, and/or runny nose, with or without eating adequately and normal conscious level, and with the (d) following past medical history and (e) combination of symptoms — what would be the most appropriate next step (f)? (Rate on scale 1–9: 1 = inappropriate; 9 = appropriate):

**(d) Past medical history**	**(e) Symptoms**						**(f) Next step? Three options:**	**Rate the appropriateness by age (scale score 1–9: 1 = totally inappropriate and 9 = totally appropriate)**		
	**1. Ear pain**	**2. Wheezing or ‘noisy breathing’**	**3. Shortness of breath**	**4. Reduced fluid intake**	**5. Less active or socially interactive than usual**	**6. High fever**	**1. Monitor, home care, go to pharmacy, or routine (non- urgent) GP appointment**	**(a) 12–23 months**	**(b) 24 months–4 years and 11 months**	**(c) 5–12 years**
							**2. Same-day GP if daytime or 111 for out-of-hours appointment**			
							**3. Go to ED or telephone 999**			
None (previously well aside from usual childhood illnesses)							1			
							2			
							3			
Previous admission for asthma, bronchiolitis, or other respiratory condition							1			
							2			
							3			

a This scenario shows a situation of only one symptom, denoted by the yellow fill for symptom 1 — ear pain.

Supplementary Table S1 summarises the results of round 2 ratings. Supplementary Table S2 shows the number of scenarios in which each next step was considered appropriate by the number of symptoms present. Increasing numbers of symptoms were generally associated with escalation of care from home care to NHS 111/GP to ED/999. Past medical history of hospital admission for asthma, bronchiolitis, or other respiratory condition had a nominal impact on panel ratings. The threshold for accessing healthcare services generally increased with the age of the child.

### Home care (with or without pharmacy services)

Panellists agreed that home care, with or without the support of a pharmacist, is appropriate for the children described in the clinical stem of the scenario. Additionally, the panel rated home care appropriate after this initial timeframe, where the child has ear pain only (irrespective of age) and in children aged ≥5 years in the presence of ear pain and reduced fluid intake or presenting as less socially interactive than usual (Box 2).

Supplementary Table S3 shows that ear pain, being less socially interactive than usual, reduced fluid intake, and high fever were rated by the panel as being most likely to be suitable for home care if they are the only symptoms present.

**Box 2.** Scenarios rated by panellists as appropriate to consider for home care^a^

**Past medical history (d)**	**Age**		
	**(a) 12–23 months**	**(b) 24 months–4 years and 11 months**	**(c) 5–12 years**
None (previously well aside from usual childhood illnesses)	If the child only has ear pain^b^		
	No consensus/normal parental decision making required^d^		If the child only has reduced fluid intake for ≤24 hours
			If the child only less socially interactive than usual
			If the child has only had a high fever for ≤24 hours^c^
			If the child only has ear pain^c^ and reduced fluid intake OR is less socially interactive than usual
Previous admission for asthma, bronchiolitis, or other respiratory condition	No consensus/normal parental decision making required^d^	If the child only has ear pain^b^	

a *After considering the clinical stem, which states: for the parent of a child aged (a) 12–23 months/(b) 24 months–4 years and 11 months/(c) 5–12 years with* ≥*1 week of respiratory infection symptoms, for example, cough, sore throat, and/or runny nose, with or without eating adequately and normal conscious level, and with the (d) following past medical history and (e) combination of symptoms — what would be the most appropriate next step (f) (Rate on scale 1–9 = 1 inappropriate; 9 = appropriate) (see Box 1).*

b *For children who have not been on a recent flight, this is nearly always due to infection and that ear pain can take* ≤*8 days^24^ for the symptom to fully resolve in the majority of children.*

c This guidance also applies to children who have previously been admitted for asthma, bronchiolitis, or another respiratory condition.

d Where no consensus could be reached, the process was essentially unable to add anything to parents’ routine decision making, which therefore returns to being the default position.

### NHS 111 or same-day GP appointment

Box 3 shows that accessing GP services was an appropriate next step in the presence of symptoms in children aged <5 years, in particular those aged between 12–23 months, in the presence of multiple symptoms, with the exception of high fever in isolation. Accessing the GP was generally seen as appropriate for children with shortness of breath alone in all age groups.

**Box 3:** Scenarios rated by panellists as may be appropriate to attend the GP^a^

**(d) Past medical history**	**Age**		
	**(a) 12–23 months**	**(b) 24 months–4 years and 11 months**	**(c) 5–12 years**
None (previously well aside from usual childhood illnesses)	If the child only has shortness of breath^b^		
	If the child only has high fever^c^	If the child only has wheezing^c^	No consensus was agreed/normal parental decision making required^d^
	If the child has ear pain and high fever^c^		
	If the child has any symptoms combined with shortness of breath, wheeze, or high fever		
	Any combination of 3 symptoms^c^	Any combination of 3 symptoms apart from: high fever, reduced fluid intake, and shortness of breath^c^	
	Combinations of 4 symptoms, where this does not include wheezing AND/OR shortness of breath^b,c^		
	If the child has 5 symptoms that do not include shortness of breath or wheezing^b^		
Previous admission for asthma, bronchiolitis, or other respiratory condition	If the child only has wheezing	If the child only has high fever	If the child only has wheezing
	If the child has any symptoms combined with shortness of breath, wheeze, or high fever	No consensus was agreed/normal parental decision making required^d^	If the child has any symptoms combined with shortness of breath, wheeze, or high fever
	Any combination of 3 symptoms apart from: less socially active than usual, high fever, and shortness of breath^b^		
	All combinations of 4 symptoms apart from: reduced fluid intake, less active or socially interactive than usual, high fever, shortness of breath^b^		
		AND reduced fluid intake, high fever, wheezing, and shortness of breath^b^	
	If the child has 5 symptoms, but does not have wheezing^b^	If the child has 5 symptoms that do not include shortness of breath or wheezing^b^	

a *After considering the clinical stem, which states: for the parent of a child aged (a) 12–23 months/(b) 24 months–4 years and 11 months/(c) 5–12 years with* ≥*1 week of respiratory infection symptoms, for example, cough, sore throat, and/or runny nose, with or without eating adequately and normal conscious level, and with the (d) following past medical history and (e) combination of symptoms — what would be the most appropriate next step (f) (Rate on scale 1–9 = 1 inappropriate; 9 = appropriate) (see Box 1).*

b ED was generally deemed more appropriate in most circumstances.

c This guidance also applies to children who have previously been admitted for asthma, bronchiolitis, or another respiratory condition.

d Where no consensus could be reached, the process was essentially unable to add anything to parents’ routine decision making, which therefore returns to being the default position. ED = emergency department.

Supplementary Table S4 shows that combinations of ≥2 symptoms in addition to the clinical stem were generally seen as appropriate for NHS 111 or same-day GP appointment, particularly where the child has shortness of breath, wheeze, or a high fever.

### Emergency department and 999

Box 4 shows the scenarios in which the panel deemed it appropriate (in most circumstances) to seek assessment in the ED. The scenarios are generally composed of ≥3 symptoms, unless this is a combination of the two most heavily weighted symptoms for the youngest age group (shortness of breath combined with wheeze). Additionally, it was deemed appropriate if the child has only shortness of breath, is aged 5–12 years, and has a past medical history of being admitted for a respiratory illness.

**Box 4.** Scenarios rated by panellists as may be appropriate to attend emergency department^a^

**Past medical history (d)**	**Age**		
	**(a) 12–23 months**	**(b) 24 months–4 years and 11 months**	**(c) 5–12 years**
None (previously well aside from usual childhood illnesses)	If the child has shortness of breath combined with wheeze		
	If the child is less active or socially interactive than usual, has high fever, and reduced fluid intake	No consensus/normal parental decision making required^c^	
	If the child has ear pain, is less active or socially interactive than usual, has a high fever, and is wheezing or has ‘noisy breathing’^b^		
	Any four symptom combinations that combine both wheeze and shortness of breath^a^		No consensus/normal parental decision making required^c^
	All symptoms	All symptoms^b^	
	If the child has high fever and reduced fluid intake combined with wheeze OR shortness of breath^b^		
	If the child has ear pain, high fever, and shortness of breath^b^		
	If the child is less active or socially interactive than usual, has high fever, and combined with wheeze OR shortness of breath		
	If the child has high fever, combined with shortness of breath and wheeze^b^		
	Reduced fluid intake, less active or socially interactive than usual, high fever, and wheeze OR shortness of breath^b^		
	If the child has ear pain, reduced fluid intake, high fever, and shortness of breath^b^		
	All five symptom combinations^b^		
Previous admission for asthma, bronchiolitis, or other respiratory condition	No consensus/normal parental decision making required^c^		If the child only has shortness of breath
	If the child is less active or socially interactive than usual, has high fever, and combined with wheeze OR shortness of breath		If the child is less active or socially interactive than usual, has high fever, and combined with wheeze OR has shortness of breath except when these are combined with ear pain and being less active or socially interactive than usual

a *After considering the clinical stem, which states: for the parent of a child aged (a) 12–23 months/(b) 24 months–4 years and 11 months/(c) 5–12 years with* ≥*1 week of respiratory infection symptoms, for example, cough, sore throat, and/or runny nose, with or without eating adequately and normal conscious level, and with the (d) following past medical history and (e) combination of symptoms — what would be the most appropriate next step (f) (Rate on scale 1–9 = 1 inappropriate; 9 = appropriate) (see Box 1).*

b This guidance also applies to children who have previously been admitted for asthma, bronchiolitis, or another respiratory condition.

c Where no consensus could be reached, the process was essentially unable to add anything to parents’ routine decision making, which therefore returns to being the default position. ED = emergency department.

Supplementary Table S5 shows that symptoms most likely to be rated as appropriate for ED assessment were shortness of breath, wheeze, and high fever.

The panel identified that generally it would be appropriate for children with ≥5 symptoms to attend the ED. They were also in agreement that it would be appropriate in most circumstances for a parent of a child aged 12–23 months with all six symptoms and a past medical history of admission for a respiratory condition to call 999.

## DISCUSSION

### Summary

A representative multidisciplinary panel of professionals responsible for the care of children with RTIs used the RAND/UCLA Appropriateness Method to agree criteria to support parents deciding if, when, and where to access health services for children with ≤1 week of RTI symptoms. Scenarios took in to account the child’s age, past medical history, and symptoms. Panellists agreed that parents of an ‘average’ child with ≤1 week of respiratory infection symptoms including cough, sore throat, and/or runny nose, with or without eating adequately, with or without ear pain, and a normal conscious level would be appropriate for home care.

In this study, criteria to identify children with symptoms of RTI suitable for home care were developed and the study found that it is often appropriate for them to be managed without medical input. If implemented, these findings could support improved health care-seeking behaviour, by providing a clear and evidence-based information source, allowing pressured services to focus care where most needed.

### Strengths and limitations

To the authors’ knowledge, this is the first time any attempt has been made to establish criteria for parents to guide them in health care-seeking behaviour. The present study identified and prioritised scenarios based on best practice and in comparison with existing evidence using the validated RAND/UCLA method. Panellists represented the full range of healthcare services available to parents of children with RTIs.

All studies of this type have limitations. First, the authors would have liked to vary the presence of other symptoms such as cough, sore throat, rash, and phlegm, along with their severity and duration.^24^ However, each additional symptom exponentially increases the number of scenarios. Similarly, the present results pertain only to children aged ≥12 months because it was considered that many clinicians would advise parents of younger children to seek help if they had any concerns. Second, the method is only possible by considering the ‘average’ child. Health services and parents wishing to use these criteria need to be aware that where a child is no longer considered ‘average’, a parent’s knowledge of, and concern about, their child is paramount. Third, by providing a set number of additional symptoms, it is possible that panel members would have automatically deemed a child with none to be appropriate for self-management and a child with all additional symptoms in need of 999 assistance, as this would be the simplest way to approach the high volume of difficult decisions they were being asked to make. Finally, this research does not provide any reassurance for parents who have children with RTI symptoms for >1 week (unless ear pain is the only additional symptom; Box 2).

### Comparison with existing literature

The present study builds on work of the HAPPY AUDIT by providing greater insight into RTI symptoms in children and decision making before accessing the GP.^25^ Previous studies have attempted to modify help-seeking behaviour through parental education,^26^ but the evidence provided within these resources was not developed using RAM or developed using a panel representing a full range of healthcare professional expertise. The interventions developed were primarily based on the experiences of authors,^27^^–^^29^ or through the convening of a multidisciplinary group,^30^ with the booklets developed then being reviewed by parents and other professionals.^28^^,^^29^ One study developed an instruction booklet for parents of a child with acute otitis media by surveying 12 physicians to develop a criteria of essential information.^31^ Criteria were included in the final booklet if they were selected as being appropriate by >75% of the physicians.^31^

### Implications for research and practice

Clinicians and policymakers may wish to use and promote criteria presented in this study to parents to help inform health care-seeking decision making. Any symptom may be regarded as abnormal and therefore requiring treatment; these criteria could be considered useful to distinguish ‘normal’ from ‘abnormal’ infections. The authors think there are three areas of fruitful future research. First, the authors have conducted a co-design event with parents, GPs, pharmacists, 111 staff, and ED consultants to consider the most user-friendly modes of result dissemination and the potential for a decision-support resource for parents, which will be reported separately. The criteria have face validity based on expert professional opinion and available evidence.^13^ However, this is a minimum prerequisite for a quality measure and subsequent development work would be required to provide empirical evidence of higher types of validity as well as reliability, acceptability, and other measures.^13^ Second, prospective cohort methods could be used with parents recording symptoms in children to confirm the appropriateness of help-seeking behaviour suggested by the present findings. Third, this method could be scaled up to provide help-seeking criteria to a broader range of conditions and scenarios where there is evidence of uncertainty regarding the appropriateness of current healthcare use.
